# Effects of Catecholamine Stress Hormones Norepinephrine and Epinephrine on Growth, Antimicrobial Susceptibility, Biofilm Formation, and Gene Expressions of Enterotoxigenic *Escherichia coli*

**DOI:** 10.3390/ijms242115646

**Published:** 2023-10-27

**Authors:** Lingdi Niu, Mingchun Gao, Shanshan Wen, Fang Wang, Haikun Shangguan, Zhiyuan Guo, Runxiang Zhang, Junwei Ge

**Affiliations:** 1 College of Veterinary Medicine, Northeast Agricultural University, Harbin 150030, China; 2 Heilongjiang Provincial Key Laboratory of Zoonosis, Harbin 150030, China; 3 State Key Laboratory of Veterinary Biotechnology, Harbin Veterinary Research Institute, Chinese Academy of Agricultural Sciences, Harbin 150069, China; 4 College of Animal Science and Technology, Northeast Agricultural University, Harbin 150030, China

**Keywords:** ETEC, catecholamine hormone, transcriptome analysis

## Abstract

Enterotoxigenic *Escherichia coli* (ETEC) is a significant contributor to diarrhea. To determine whether ETEC-catecholamine hormone interactions contribute to the development of diarrhea, we tested the effects of catecholamine hormones acting on ETEC in vitro. The results showed that in the presence of norepinephrine (NE) and epinephrine (Epi), the growth of 9 out of 10 ETEC isolates was promoted, the MICs of more than 60% of the isolates to 6 antibiotics significantly increased, and the biofilm formation ability of 10 ETEC isolates was also promoted. In addition, NE and Epi also significantly upregulated the expression of the virulence genes *feaG*, *estA*, *estB*, and *elt*. Transcriptome analysis revealed that the expression of 290 genes was affected by NE. These data demonstrated that catecholamine hormones may augment the diarrhea caused by ETEC.

## 1. Introduction

In developing countries, enterotoxigenic *Escherichia coli* (ETEC) is a significant contributor to diarrhea in children [1], and many of them are shown to carry the organism asymptomatically in their gut [2]. Some studies have shown that it remains a major cause of travelers’ diarrhea, which occurs in people visiting or returning from ETEC-endemic regions [3]. Severe watery diarrhea with fast dehydration and prostration within a few hours are the clinical manifestations of ETEC infection [4]. ETEC is also a major cause of diarrhea in animals, especially post-weaning diarrhea (PWD). It is one of the most common diseases among piglets. ETEC that causes PWD usually bears virulence factors such as F4 (K88) or F18 adhesins [5]. The ETEC infection mechanism relies on its fimbria adhesions, which initiate ETEC colonization and promote enterotoxin secretion by interacting with the brush border of enterocytes [6]. ETEC infection also brings about diarrhea and influences immune responses [7,8,9], tight-junction function [10,11,12,13], autophagy [14], and other intestinal functions.

In 1992, Lyte and Ernst [15] assessed the effect of bacterial growth under stress hormones and came up with the concept of “microbial endocrinology”. They showed a two-way interaction between microorganisms and human neuroendocrine factors. It was shown in past research that stress could increase neuroendocrine hormones, particularly glucocorticoids and catecholamines. The release of some hormones, such as catecholamines, can affect the homeostatic balance of the body and influence the course of numerous diseases [16]. Catecholamines have also been confirmed to promote and enhance some bacterial growth ability and virulence, thereby increasing the susceptibility of the host to bacterial microorganisms and affecting the interaction of bacterial microorganisms with hosts in the disease process [17,18]. Norepinephrine (NE) and epinephrine (Epi) have a good growth-promoting effect on most bacteria, including *Listeria monocytogenes* [19], *Streptococcus pneumoniae* [20], *Aeromonas hydrophila* [21], *Campylobacter jejuni*, and various *Vibrio* spp. [22]. Some studies have also shown that the bacteria’s virulence and biofilm-formation ability, such as those of *Salmonella* sp. [23] and EHEC [24], can be regulated by catecholamines, thereby affecting the infection outcome of these bacteria in numerous hosts. Given that hosts can carry ETEC in their guts asymptomatically, catecholamine hormones may play a significant role in the development of ETEC-related diseases when pressure occurs. However, the mechanism by which catecholamine hormones affect ETEC-host interactions is unclear. In this study, our aim was to determine whether, when exposed to catecholamine hormones, ETEC would show an increase in the pathogenicity factors likely the antimicrobial susceptibility, biofilm formation ability, and bacterial virulence gene expression, which could potentially solve the problem of disease exacerbation caused by ETEC under stress conditions. Our data provide valuable insights into the role of stress-related catecholamines in the effects of ETEC.

## 2. Results

### 2.1. NE and Epi Promote Growth of F4^+^ ETEC

**Figure 1:**
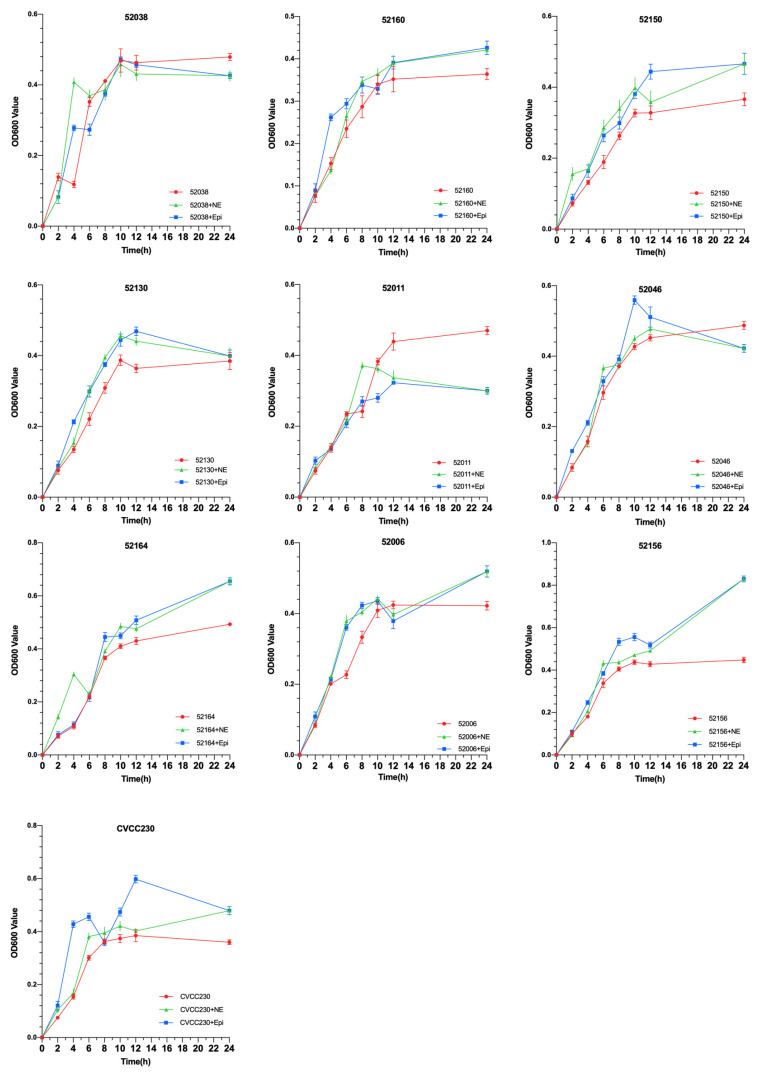
Impact of norepinephrine (NE) and epinephrine (Epi) on the growth of ten isolates of F4^+^ enterotoxigenic *Escherichia coli* (ETEC).

To explore the effects of NE and Epi on the growth of 10 F4^+^ isolates, growth curves were drawn using the OD600 values of each group. As shown in Figure 1, under the effects of NE and Epi, respectively, the growth of 80% F4^+^ ETEC was obviously promoted after 6 h. The results also showed that NE and Epi had little effect on the growth of the 52038 isolate, while NE and EPI inhibited the growth of the 52011 isolate. As shown in Supplementary Figures S1 and S2, in the ETEC+NE+α and ETEC+Epi+α groups, the bacterial density was almost the same at 12 and 24 h compared with the ETEC+α group. However, in the 52038, 52160, and 52130 isolates, compared with the ETEC+Epi group, the bacterial density in the ETEC+Epi+β antagonist-added group was significantly lower than that in the group without antagonists. Thus, Epi may promote growth through the β-adrenergic receptor antagonist.

### 2.2. NE and Epi Enhance Antimicrobial Susceptibility of F4^+^ ETEC

**Figure 2:**
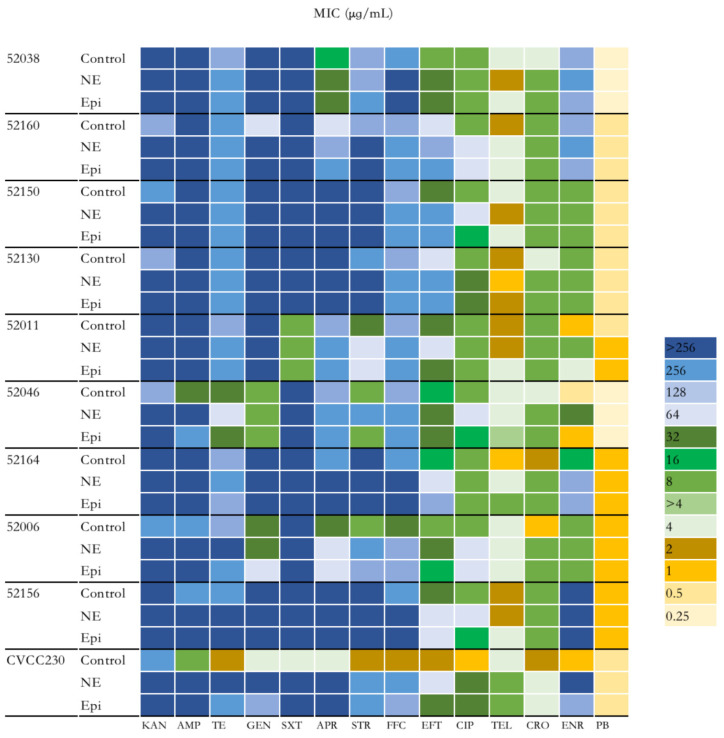
Impact of NE and Epi on MIC of F4^+^ ETEC to 14 antibiotics. MIC, minimum inhibitory concentration. KAN, kanamycin; AMP, ampicillin; TE, tetracycline; GEN, gentamicin; SXT, sulfamethoxazole/trimethoprim; APR, apramycin; STR, streptomycin; FFC, florfenicol; EFT, ceftiofur CIP, ciprofloxacin; TEL, tylosin; CRO, ceftriaxone; ENR, enrofloxacin; PB, polymyxin B.

To explore the effect of NE and Epi on the antimicrobial susceptibility of F4^+^ ETEC isolates, 14 antibiotics were investigated. As shown in Figure 2 and Supplementary Table S1, the group of ETEC with NE had higher antimicrobial susceptibility than those without. Overall, except for polymyxin B, the MIC of the remaining 13 antibiotics to CVCC230 isolate was obviously increased by NE and Epi compared with the control group. According to the antimicrobial susceptibility test of the isolates, nine F4^+^ ETEC isolates were multi-drug-resistant isolates. It is worth noting that under the action of NE and Epi, the MIC to AMP against 52046 isolate was increased from 32 μg/mL to >256 μg/mL and 256 μg/mL; the MIC of NE and Epi to GEN against 52160 isolate was increased from 64 μg/mL to >256 μg/mL; Epi increased the MIC of APR against 52160 isolate from 64 μg/mL to 256 μg/mL; NE increased the MIC of STR against 52046 and 52006 isolates from 8 μg/mL to 256 μg/mL, while Epi increased the MIC of STR against 52006 isolate from 8 μg/mL to 128 μg/mL; under the action of NE and Epi, the MIC to FFC against 52006 isolate was increased from 32 μg/mL to 128 μg/mL; NE increased the MIC of EFT against 52038, 52150, 52130, 52164, and 52006 isolates while Epi increased the MIC against 52038, 52160, 52150, 52130, and 52164 isolates; NE also increased the MIC of CIP against 52038, 52150, 52130, 52046, 52006, 52156 isolates, and Epi increased the MIC against 52038, 52130, and 52006 isolates; the MIC of NE and Epi to CRO against 52164 isolate was increased from 2 μg/mL to 8 μg/mL; NE increased the MIC of ENR against 52011, 52046 and 52164 isolates while Epi increased the MIC against 52011 and 52164 isolates.

### 2.3. NE and Epi Promote Biofilm Formation of F4^+^ ETEC

**Figure 3:**
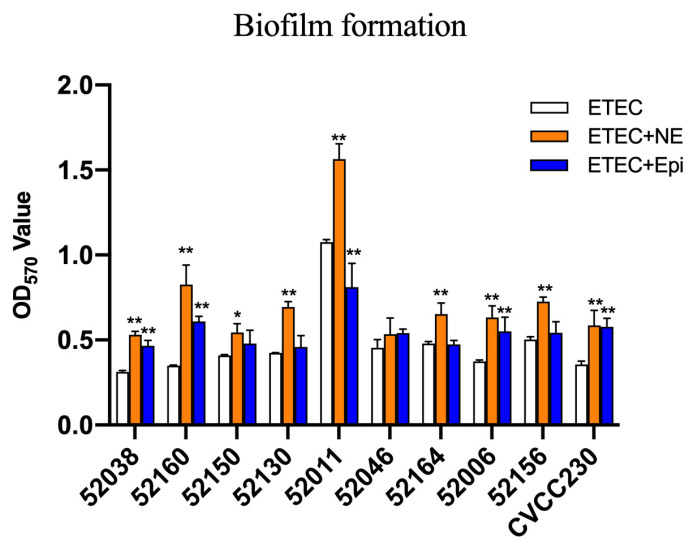
Impact of NE and Epi on biofilm formation by ETEC. The ordinate represents optical density (OD), which was measured at 570 nm. Results are the mean ± SD from 3 independent experiments (each with *n* = 3), * *p <* 0.05, ** *p <* 0.01.

The microtiter-plate test was used to measure the ability of ETEC biofilm formation. The results in Figure 3 after 24 h showed that 52038, 52160, 52150, 52006, and CVCC230 isolates were unable to form biofilm. 52130, 52046, 52164, and 52156 isolates were weak biofilm producers, whereas the 52011 isolate was a moderate biofilm producer. While the culture medium contained NE, it strongly increased biofilm biomass, showing that 52038, 52150, 52130, 52046, 52164, 52006, and CVCC230 isolates were able to form weak biofilm, 52160 and 52156 isolates formed moderate biofilm, and 52011 isolates formed strong biofilm. In the presence of Epi, except for the 52011 isolate, which was a moderate biofilm producer, the other isolates were weak biofilm producers. Thus, the addition of NE and Epi led to a strongly increased biofilm biomass.

### 2.4. NE and Epi Enhance Virulence-Related Gene mRNA Expression Levels of F4^+^ ETEC

**Figure 4:**
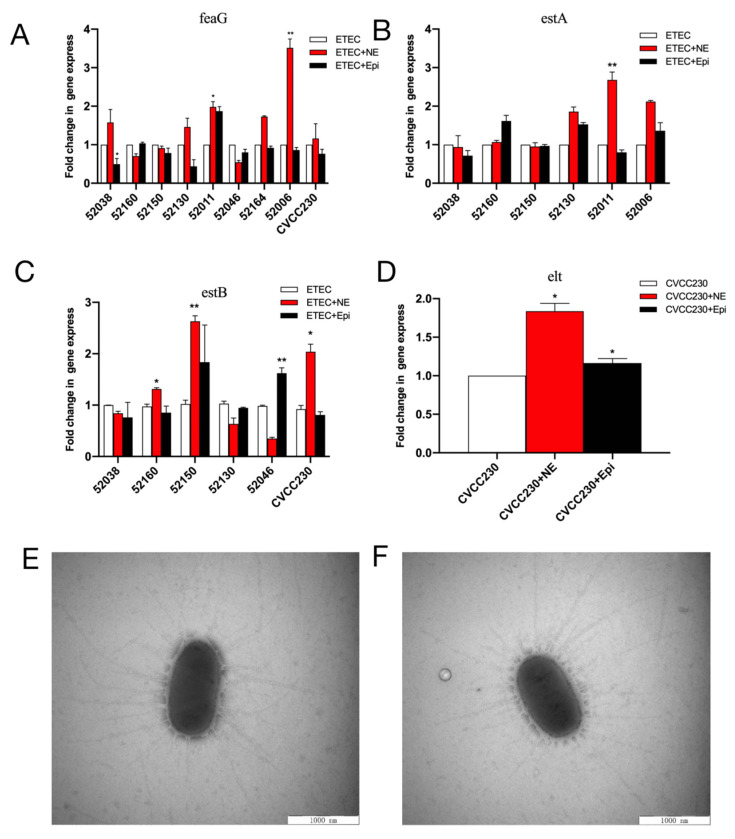
(**A**–**D**) Relative expression of the *feaG*, *estA*, *estB*, and *elt* genes in NE and Epi. Real-time qPCR reactions were used for detection, and *gapA* was seen as reference gene. The abscissa represents groups, and the ordinate represents 2^−ΔΔCT^ value. Results are the mean ± SD from 3 independent experiments (each with *n* = 3), * *p <* 0.05, ** *p <* 0.01. (**E**) Control group (CVCC230). Images obtained by TEM show fimbriae of ETEC CVCC230. (**F**) NE group (NE+CVCC230). Images obtained by TEM show fimbriae of ETEC CVCC230 in the presence of NE.

Quantitative PCR (qPCR) was used to examine the effects of NE and Epi on the mRNA expression of virulence-related genes in F4^+^ ETEC isolates. NE increased the *feaG* gene mRNA expression in 52038, 52130, 52011 (*p* < 0.05), 52006 (*p* < 0.01), and CVCC230 isolates, significantly increasing it in the 52006 isolate. Meanwhile, Epi increased it in the 52011 isolate (Figure 4A). As shown in Figure 4B, NE increased the *estA* gene mRNA expression in the 52130, 52011 (*p* < 0.01), and 52006 isolates, whereas Epi increased it in the 52160, 52130, and 52006 isolates. As shown in Figure 4C, NE increased the relative expression of the *estB* gene in 52160 (*p* < 0.05), 52150 (*p* < 0.01), and CVCC230 isolates, whereas Epi increased it in 52150 and 52046 (*p* < 0.01) isolates. NE and Epi significantly promoted the expression of the *elt* gene in the CVCC230 isolate (*p* < 0.05) (Figure 4D).

### 2.5. NE Has No Significant Effect on Fimbriae of F4^+^ ETEC

Meanwhile, fimbriae were determined by electron microscopy after staining to increase their clarity during visualization. We randomly selected five cells of ETEC CVCC230 under TEM, and the average number of fimbriae for the control (Figure 4E) and NE group (Figure 4F) were 31 ± 4.11 and 32 ± 6.5, respectively. No statistical difference existed between the two groups, indicating that NE had no obvious effect on the fimbriae of the CVCC230 isolate.

### 2.6. NE Modulates the Gene Expression Profile of F4^+^ ETEC

**Figure 5:**
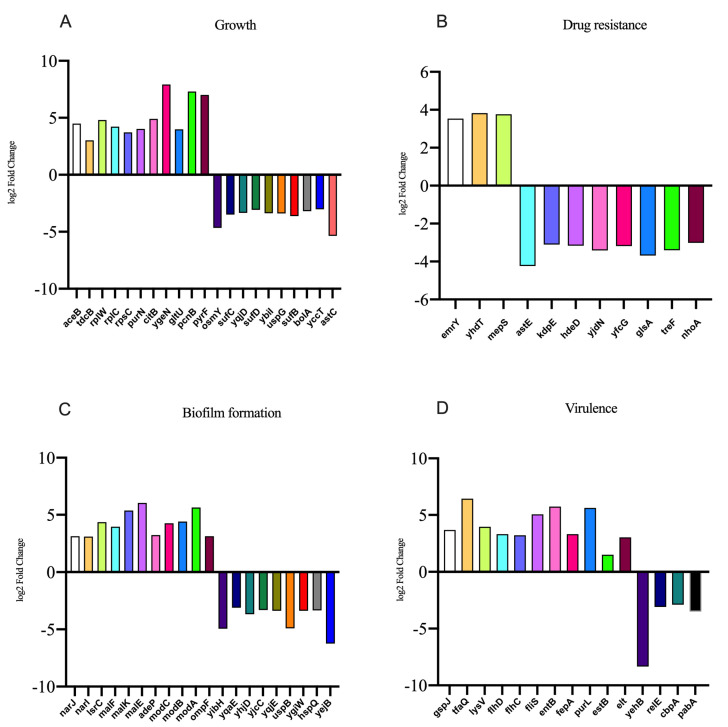
Results of some differentially expressed genes related to the (**A**) growth, (**B**) drug resistance, (**C**) biofilm formation, and (**D**) virulence of CVCC230 isolates after adding NE.

**Figure 6:**
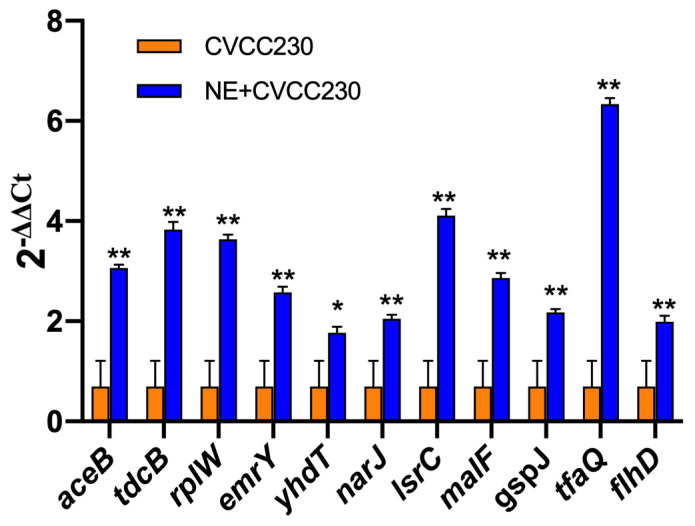
Quantitative RT-PCR analysis of selected genes to verify the transcriptome data. Real-time qPCR reactions were used for detection, and *gapA* was seen as reference gene, and the relative expression level of each gene was calculated by the comparative 2^−ΔΔ^*^CT^* method. Results are the mean ± SD from 3 independent experiments (each with *n* = 3), * *p* < 0.05, ** *p* < 0.01.

In the presence of NE, the number of genes with upregulated expression in the F4^+^ ETEC CVCC230 isolate transcriptome is depicted in Figure 5. Results showed the differential expression of 290 genes, among which 162 genes were upregulated and 128 genes were downregulated. The expression of genes related to the growth of the F4^+^ ETEC CVCC230 isolate was upregulated by 74 genes and downregulated by 21 genes. Figure 5A (Supplementary Table S2) shows that part of the more pronounced differences in expression. Figure 5B (Supplementary Table S3) shows that the expression of genes related to the resistance of the F4^+^ ETEC CVCC230 isolate was upregulated by 3 genes and downregulated by 8 genes. The expression of genes related to the biofilm formation of the F4^+^ ETEC CVCC230 isolate was upregulated by 31 genes and downregulated by 24 genes. Figure 5C (Supplementary Table S4) shows that this is one of the more pronounced differences in expression. Figure 5D (Supplementary Table S5) shows that the expression of genes related to the virulence of the F4^+^ ETEC CVCC230 isolate was upregulated by 11 genes and downregulated by 4 genes. To verify the RNA sequencing data, several selected genes (*aceB*, *tdcB*, *rplW*, *emrY*, *yhdT*, *narJ*, *lsrC*, *malF*, *gspJ*, *tfaQ*, and *flhD*) were confirmed by qRT-PCR. The mRNA expression levels of these genes (Figure 6) were consistent with those determined from the transcriptome sequencing data (Figure 5), suggesting that the DEG database obtained from transcriptional sequencing is reliable and may be further studied.

**Figure 7:**
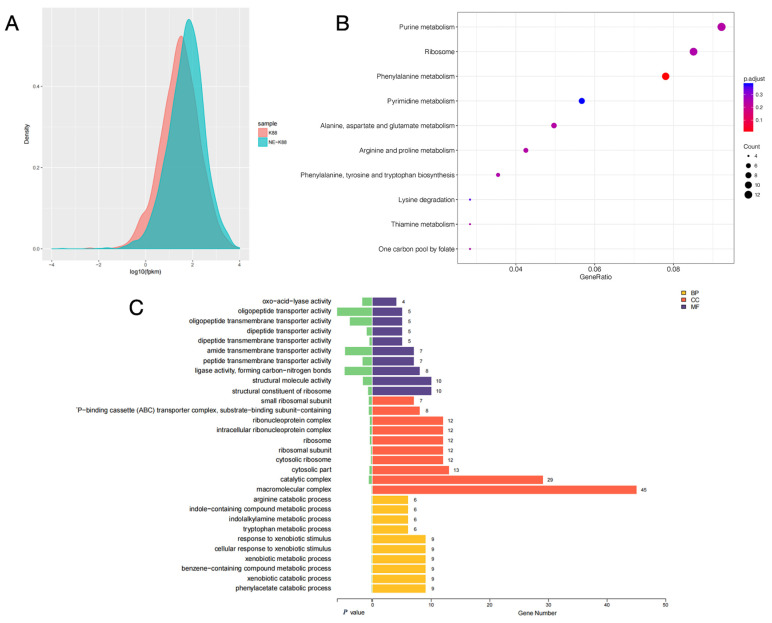
(**A**) fragments per kilobase of exon model per million mapped fragments (FPKM) density distribution comparison chart. The horizontal coordinate is log10 FPKM; the higher the value, the higher the gene expression. The vertical coordinate is the gene density. Each color in the graph indicates one sample. (**B**) The horizontal coordinate is GeneRatio, which indicates the ratio of the number of differentially expressed mRNAs in the pathway entry to the total number of differentially expressed genes, and the larger the value of GeneRation, the higher the enrichment of differentially expressed mRNAs in the KEGG pathway. The vertical coordinate is −log10 (Q-value). The larger the value of −log10 (Q-value), the higher the enrichment of differentially expressed mRNAs in the KEGG pathway. The closer the entry to the upper right corner of the graph, the higher the enrichment and the higher the reference value; conversely, the closer the entry to the lower left corner, the lower the reference value. (**C**) Differential gene ontology (GO) enrichment bar graph. Different colors represent different GO functional classes, and the number at the end of each bar represents the number of genes enriched in that GO category and the number of differential genes enriched in that GO category. Cellular component (CC), molecular function (MF), and biological process (BP). The “^-^P–binding cassette (ABC) transporter complex” means “ATP–binding cassette (ABC) transporter complex”.

The gene expression levels of samples from CVCC230 and CVCC230+NE groups were compared by fragments per kilobase of exon model per million mapped fragments (FPKM) distribution plots of all genes, and the final FPKM was the average of all replicates, and the results are shown in Figure 7A. The results showed that the gene expression of the CVCC230+NE group was higher than that of the CVCC230 group. Figure 7B showed that NE acting on ETEC may mainly affect genes related to purine metabolism, ribosome, and phenylalanine metabolism. The bar chart of differential gene ontology (GO) enrichment can visually reflect the distribution of the number of target genes in the enriched GO functions. Figure 7C shows the most significant (*p*-value level) 10 GO functions enriched in biological process (BP), cellular component (CC), and molecular function (MF). The genes affected by NE acting on ETEC were more enriched in the direction of macromolecular complexes.

## 3. Discussion

Some studies have indicated that numerous diseases are closely related to stress [16]. Under stress, the release of catecholamines can increase 20–40 times, up to 0.17–0.54 μg/min [25]. Catecholamine stress hormones have been shown to promote bacteria’s growth and virulence in vitro through multiple mechanisms, as demonstrated in previous studies on microbial endocrinology [26,27]. The diarrhea in weaned piglets is closely related to stress; therefore, we chose nine ETEC isolates that were isolated from piglets with diarrhea after weaning stress to investigate the effect and mechanism of stress hormones on ETEC. Here, we focused on the stress-signaling molecules NE and Epi, which could enhance the growth, antimicrobial susceptibility, biofilm formation, and virulence of ETEC in vitro. This may be due to the fact that NE modulates the gene expression of ETEC. Based on gaps in microbial endocrinology research, we hope to investigate the relationship between catecholamine hormones and bacteria and advance microbial endocrinology research through our studies. Future research should focus on the mechanisms underlying the promotion of ETEC growth and pathogenicity to solve diarrhea caused by ETEC.

When the F4^+^ ETEC isolates were tested in vitro in the presence of NE and Epi, 9 out of 10 isolates showed a significantly increased growth rate. On the one hand, the two adrenergic receptors, phentolamine and propranolol, may be involved in such enhancement (Figure 1). Previous studies have used catecholamine-receptor antagonists to investigate the effect of catecholamines on staphylococci [28]. On the other hand, the promotion could be related to the differential expression of *aceB*, *tdcB*, *rplW*, and *rplC* genes, among others (Figure 5A). The *aceB* gene encodes *E. coli* malate synthase A, one of the key enzymes of the glyoxylate cycle (Gene ID: 948512); the *tdcB* gene is catabolic threonine dehydratase; it is one of several enzymes carrying out the first step in serine degradation (Gene ID: 947633); the *rplW* gene is 50S ribosomal subunit protein L23; it can provide a chaperone docking site that links protein biosynthesis with protein folding (Gene ID: 947819); the *rplC* gene is 50S ribosomal subunit protein L3 (Gene ID: 947817). According to some in vitro studies, NE caused growth in the bacterial populations of pathogenic EHEC [29] and *Salmonella* Typhimurium [30]. In a murine model of trauma-induced norepinephrine release in vivo, there was an increase in the total gram-negative population, most notably *E. coli* [31]. Our findings also showed that NE and Epi inhibited the growth of the F4^+^ ETEC 52011 isolate, which is supported by other studies, such as NE inhibiting the growth of *Prevotella intermedius* [32] and *Porphyromonas pulposus* [33]. Sturbelle et al. also found that the use of Epi at a concentration of 50 μM had little effect on the growth kinetics of F4^+^ ETEC E68 [34]. The F4^+^ ETEC isolates responded differently to NE and Epi, which may be related to the concentration of hormones [32,33,35,36], types of hormones, different strains of bacteria [37], and content of iron in the culture medium [25,38]. The possible reason is that NE/Epi can accelerate the bacterial acquisition of iron through siderophore-like properties, thereby promoting bacterial growth in specific environments, and the use of different media may have different effects on bacterial growth. This study used as many ETEC strains as possible to demonstrate that catecholamine hormones can modulate the growth and characteristics of most ETEC strains. The MICs of more than 60% of the isolates to six antibiotics significantly increased, and more than 90% of ETEC isolates showed increased MICs to florfenicol and ceftiofur due to the presence of NE and Epi (Figure 2), and the MIC values obtained from the three replications conducted in this study indicated consistency. The increased antimicrobial susceptibility may be associated with differential expression of some drug-resistance-related genes, such as *emrY*, *yhdT*, *mepS*, and *astE* genes (Figure 5B). In the next step, we will determine the role of these genes in the resistance mechanism. The *emrY* gene is the tripartite efflux pump membrane subunit EmrY, which is a putative multidrug efflux protein belonging to the major facilitator superfamily (MFS) of transporters (Gene ID: 946835); the *yhdT* gene is the DUF997 domain-containing protein YhdT, which has a role in drug resistance mechanisms (Gene ID: 947762); and the *mepS* gene is peptidoglycan endopeptidase (Gene ID: 946686); the *astE* gene is succinylglutamate desuccinylase (Gene ID: 946256). In the livestock industry, antibiotics have been used for decades to combat diarrhea problems in weaned piglets and promote growth [39]. That is consistent with previous observations that NE increases the multi-antimicrobial susceptibility of *Acinetobacter baumannii* to tigecycline resistance [40]. Additionally, Freestone et al. discovered that the catecholamine inotropes used in the clinical setting may help antibiotic-damaged staphylococci recover and thrive [28]. Cuvas Apan et al. also found that drug resistance in *E. coli* and *Pseudomonas aeruginosa* increased when stimulated by Epi, NE, and dopamine [36]. The low influence of polymyxin B on the MIC of ETEC isolates in the presence of NE and Epi may be explained by the fact that these bacteria are more sensitive to polymyxin B, and the F4^+^ ETEC isolate was first killed by polymyxin B before they interacted. This finding is consistent with other previous observations that NE has no significant influence on the effect of polymyxin B on the multi-drug-resistant *A. baumannii* [40]. Epi also attenuated the viability of *S.* Typhimurium to polymyxin B treatment [41]. Past research has shown that two genes associated with antimicrobial resistance (Cj1583c and Cj0193c) are overexpressed in *C. jejuni* at 100 μM Epi or NE treatment [42].

Here, we demonstrated that NE was able to significantly promote the biofilm formation of 10 ETEC isolates, and Epi was able to promote the great majority of ETEC biofilm formation apart from the 52011 isolate (Figure 3). The reason for the promotion may be related to the differential expression of *narJ*, *narI*, *lsrC*, *malF*, and *malK* genes, etc. (Figure 5C). The *narJ* gene is a nitrate reductase 1 molybdenum cofactor assembly chaperone (Gene ID: 945807), and the *narI* gene is a nitrate reductase A subunit gamma (Gene ID: 945808), *NarJ* can block membrane attachment via *narI* until activation is complete, and *narI* is involved in localizing NarGHI to the membrane induced by anaerobiosis plus nitrate; the *lsrC* gene is the Autoinducer-2 ABC transporter membrane subunit; it is a membrane component of a predicted ATP-dependent sugar transporter (Gene ID: 946105); the *malF* gene is the maltose ABC transporter membrane subunit (Gene ID: 948532); the *malK* gene is the maltose ABC transporter ATP binding subunit (Gene ID: 948537). This finding was consistent with recent work showing that catecholamines promote the formation of biofilm in vitro in *S. pneumoniae* [20,43], *Vibrio javier* [44], *Yersinia ruckeri* [45], *Enterococcus faecalis* [26], and gram-positive strains such as *Cutibacterium acnes* [27]. Biofilms play an important role in improving microbial resistance to antibiotics [46]. Studies have shown that the resistance of microorganisms forming biofilms to most antibiotics is much higher than that during planktonic growth [40]. Notably, we observed that the effects of the same dose of NE and Epi on the ability of biofilm formation and disintegration varied among different isolates. This result may be related to the specific mechanisms of the isolate, such as its different sensitivity to NE and Epi. Some studies have shown that increased or decreased biofilm formation depends on the tested strains [47], so exploring the mechanism of action of catecholamine hormones on biofilms is necessary. The formation and maintenance of biofilms are reportedly affected by many factors, such as binding nutrient sites, secretion of inhibitory molecules, intercellular communication, and contact-dependent growth inhibition [48]. Therefore, studying the effect of catecholamine hormones on ETEC’s biofilm formation is important for the spread of ETEC resistance because it helps better understand the interaction among bacteria, catecholamine hormones, and the host.

The results of this research have shown that NE and Epi can significantly upregulate the expression level of the virulence genes *feaG*, *estA*, *estB*, and *elt* (Figure 4A–D). Our transcriptome sequencing results for CVCC230 isolate were consistent with the qPCR results. It also showed that *gspJ*, *tfaQ*, *flhD*, and *flhC* genes (encoding for functions related to virulence) were regulated by NE, which may be caused by catecholamine hormones enhancing the expression of F4^+^ ETEC virulence genes (Figure 5D). The *gspJ* gene is a Type II secretion system protein (Gene ID: 947832); the *tfaQ* gene is a putative tail fiber assembly protein (Gene ID: 946060); the *flhD* and *flhC* genes are DNA-binding transcriptional dual regulators; they are transcriptional activators of the flagellar regulon (Gene ID: 945442; 947280). The infection and propagation of pathogens in hosts are related to virulence factors. Understanding the types and expression levels of virulence genes in pathogenic bacteria can guide disease prevention and control. Our observations on NE and Epi acting on ETEC agree with past reports that NE and Epi upregulated the expression of the virulence [20,34] genes. In fact, Lyte et al. demonstrated in 1997 that NE dramatically increased the growth of ETEC strain B44 and the production of K99 pilus adhesin at the protein level [49,50]. Indeed, catecholamines have been shown to increase the expression of virulence-related genes of various pathogens of land animals and humans in different models of infection, such as *E. coli* [51], *C. jejuni* [52], *Typhoid bacillus* [53], and *Vibrio parahaemolyticus* [54]. Catecholamine hormones negatively regulated the toxin genes of a small number of strains, which was most likely due to ETEC’s large individual differences or to the hormone concentration. Some studies have shown that several ETEC virulence factors are modulated by iron [55]. Chen et al. suggested that the adhesion of *E. coli* O157:H7 to the epithelium may be related to enteral catecholamines [56]. On this basis, we can propose that NE-mediated invasiveness enhancement may be the result of increased adhesion of bacteria to the epithelium. Thus, the role of these divergently regulated genes in the mechanism by which catecholamine hormones enhance the expression of virulence genes in F4^+^ ETEC is meaningful to investigate.

The signal-transduction system of bacteria is mostly a two-component system. The bacterial receptor of host Epi/NE is the QseC sensor kinase, which is also part of the QseBC system. This system was previously believed to be a quorum-mediated bicomponent system involved in the regulation of EHEC flagella and motility [57]. Phosphate can be transferred into the response regulator QseB after activation of QseC, which regulates the transcription of bacterial motility genes and flagella [58,59]. Enterohemorrhagic *E. coli* O157:H7 reportedly activates the expression of virulence islands, Shiga toxin, and flagella through the NE/Epi/auto-inducible signaling molecule-3 system [60]. Notably, the interaction among NE, ETEC, and the environment in the body is very complex. NE is known to be regulated by the sympathetic nervous system and the hypothalamic–pituitary–adrenal axis (HPA) system, whereas the excessive release of various neurotransmitters (such as catecholamines and vasoactive intestinal peptides) stimulates adrenergic receptors expressed by immune cells [61]. It can also regulate the production of cytokines and antibodies in immune cells, thereby affecting the local inflammatory response. Infection with bacteria activates pro-inflammatory gene programs, such as expressing the IL-1β, IL-6, and tumor necrosis factor genes through transcription factors [62]. Other studies have also demonstrated that experimentally induced acute psychological stress can increase the circulating levels of IL-6 and IL-1β [63] and activate NF-κB and primary leukocytes in peripheral blood monocytes to increase the pro-inflammatory effect in vitro [64]. Noradrenaline, ETEC, and the body’s internal environment interact with one another in pairs.

This study simulated the internal environment in a mouse model and found that the presence of NE affected the growth, biofilm formation, antimicrobial susceptibility, and virulence gene expression of most ETEC isolates. We only attempted to analyze the mechanism through the transcriptome results. A limitation was the pathway through which it worked, and the mechanism of action was not clarified. In the next step, we will explore the relationship between the QseBC system and the interaction among NE, ETEC, and the environment in the body.

## 4. Materials and Methods

### 4.1. Bacteria and Culture Medium

The F4^+^ ETEC CVCC230 isolate was purchased from the China Institute of Veterinary Drug Control (Beijing, China), and it was continuously passaged in Luria-Bertani (LB) broth containing concentrations of nalidixic acid (Sigma-Aldrich, St. Louis, MO, USA, N8878) from 0 to 50 μg/mL at 37 °C for 24 h to induce the nalidixic acid-resistant isolate CVCC230 (Nal^r^) [65].

Nine ETEC isolates (52038, 52160, 52150, 52130, 52011, 52046, 52164, 52006, and 52156) were isolated from diarrheal feces samples from piglets with diarrhea after weaning stress. Faecal samples were plated onto MacConkey agar plates and incubated at 37 °C overnight. Triose iron agar-positive colonies from each agar plate were isolated and identified biochemically to confirm the isolation of *E. coli* strains. Four primers, gadA, chuA, yjaA, and TSPE4.C2 [66], were used to genotype these ETEC isolates into three types (A, B1, and B2) by multiplex PCR. The existence of genes encoding various toxin factors for ETEC isolates was detected by PCR. Those toxin genes included the fimbrial gene F4 and the toxins LT, EAST1, STa, STb, Stx2e, and STX1. We also identified the biofilm formation of these isolates by colorimetric microtiter plate assay [67]. And according to the Clinical and Laboratory Standards Institute procedure, the drug sensitivity experiment on the isolates was performed by the disk diffusion method (Kirby-Bauer). This information is provided in Supplementary Table S6. The primer’s information is provided in Supplementary Table S7.

The NE, Epi, phentolamine, and propranolol used in the experiment were purchased from Sigma-Aldrich (St. Louis, MO, USA). Serum-SAPI medium (MgSO_4_ 1.01 mM, KH_2_PO_4_ 1.84 mM, glucose 2.77 mM, KCl 3.35 mM, NH_4_NO_3_ 6.25 mM, adjusted to pH = 7.50, and then added 30% (*v*/*v*) bovine serum (Clark, Needham, MA, USA, FB15011)) is a medium that mimics the host’s internal milieu, and it is mostly used to investigate the interactions between catecholamine hormones and microorganisms [15,40,68,69].

### 4.2. Growth of ETEC Isolates

The growth curves were measured using the procedure described by Gao et al. [21]. In brief, freshly cultivated ten isolates (52038, 52160, 52150, 52130, 52011, 52046, 52164, 52006, 52156, and CVCC230) were mixed in 100 mL serum-SAPI medium at a 1:100 ratio, and then NE (100 μM) or Epi (100 μM) were added as the test group (ETEC+NE group and ETEC+Epi group), and freshly cultured bacteria that did not contain NE and Epi were used as the control group. The culture was incubated at 37 °C with shaking at 180 rpm. Bacterial suspensions were taken at 2, 4, 6, 8, 10, 12, and 24 h, and absorbances were measured at 600 nm with a spectrophotometer (Thermo Fisher Scientific, New York, NY, USA).

Furthermore, 100 μM phentolamine (α-adrenergic receptor antagonist) or propranolol (β-adrenergic receptor antagonist) was added to the 100 mL serum-SAPI medium, which contained 100 μM NE or Epi for bacterial culture as the ETEC+NE+α group, ETEC+NE+β group, ETEC+Epi+α group, and ETEC+Epi+β group, respectively. Added freshly cultured ETEC bacteria to serum-SAPI medium with only phentolamine or propranolol as the control experiment (ETEC+α group and ETEC+β group) to prove that whether α-adrenergic receptor antagonist and β-adrenergic receptor antagonist alone can cause growth restriction of ETEC isolates. The culture was incubated at 37 °C with shaking at 180 rpm, bacterial suspensions were taken at 12 h and 24 h, and absorbances were measured at 600 nm with a spectrophotometer. The experiment was conducted in three replicates, and then growth curves were drawn for each group based on the OD600 values.

### 4.3. Antimicrobial Susceptibility of ETEC Isolates

The measuring method used was the minimum inhibitory concentration method according to CLSI standard procedure (Clinical and Laboratory Standards Institute) [70]. The dilution technique was used to evaluate the antimicrobial susceptibility phenotypes for kanamycin (Kana), ampicillin (AMP), tetracycline (TE), gentamicin (GEN), trimethoprim-sulfamethoxazole (SXT), apramycin (APR), streptomycin (STR), florfenicol (FFC), ceftiofur (EFT), ciprofloxacin (CIP), tylosin (TEL), ceftriaxone (CRO), enrofloxacin (ENR), and polymyxin B (PB) (Solarbio, Beijing, China). Freshly cultured ETEC bacteria in serum-SAPI medium as the control group; freshly cultured ETEC bacteria in serum-SAPI medium with NE or Epi (100 μM) as the NE or Epi group. Afterward, the bacterial liquid was diluted until the concentration of the bacterial solution was 1 × 10^6^ CFU/mL, then transferred to a 96-well plate with 100 μL per well. Serially diluted the drug storage solution with serum-SAPI medium and then added 100 μL per well to the 96-well plate. The 96-well plates were incubated statically at 37 °C for 24 h. The experiment was conducted in three replicates and measured absorbance at 600 nm with a spectrophotometer to determine the MIC value.

### 4.4. Biofilm Formation of ETEC Isolates

In the presence of NE and Epi, the biofilm formation ability of ETEC was implemented by the research of Khoramian et al. with a few modifications [67]. In short, ETEC isolates were cultivated in serum-SAPI medium until the concentration of the bacterial solution was 1 × 10^6^ CFU/mL, and then NE or Epi (100 μM) were added as the test group. ETEC isolates were cultured without NE or Epi, and the sterile serum-SAPI medium served as the control group. Then transferred to a 96-well plate with 200 μL per well for bacterial biofilm culture, stationary culture at 37 °C for 24 h. Then discard the culture solution and wash the plates twice with PBS (pH = 7.2). 200 μL of 99% methanol bound to the biofilm for 20 min, then air-dry. Then 200 μL of a 1% crystal violet solution was used to dye. This reaction was stopped after 20 min by the addition of 200 μL of 33% glacial acetic acid. After an oscillation effect of 30 min, the optical density (OD) was measured at 570 nm with a spectrophotometer. According to the judgment criteria in the reference, the isolates were classified into four grades: non-biofilm producers, weak-biofilm producers, moderate-biofilm producers, and strong biofilm producers. The experiment was conducted in three replicates.

### 4.5. Virulence Genes of ETEC Isolates

On the basis of the method of Gao et al. [21], we implemented a few modifications to the detected virulence genes. The ETEC bacteria were cultured in the serum-SAPI medium, and then NE or Epi (100 μM) were added as the test groups (NE group and Epi group). ETEC bacteria cultured in serum SAPI medium alone were used as a control group. The culture was incubated at 37 °C with shaking at 180 rpm and took bacterial suspensions at 12 h to perform subsequent operations. Firstly, centrifuge at 2–8 °C to collect the thalli, discard the supernatant, and resuspend the thalli with lysozyme (10 mg/mL) (Solarbio, Beijing, China, L1080). Next, extract RNA according to the instructions on the RNA extraction kit (TransGen Biotech, Beijing, China). Then, ReverTra Ace^®^ (TOYOBO, Shanghai, China, TRT-101) was used to reverse transcribe the isolated RNA; each 40 μL reverse transcription reaction contained 2 μg RNA template. The cDNA was used for quantification and expression of virulence genes with 2×Hi SYBR Green qPCR Mix (HaiGene Biotech Co., Ltd., Harbin, China, A2250B), including *feaG* (F4), *estA* (STa), *estB* (STb), and *elt* (LT). Furthermore, we used the Applied Biosystems^®^ 7500 Real-Time PCR Systems (Analytik Jena AG, Jena, Germany) with a two-step method: stage 1:95 °C for 5 min; stage 2:95 °C for 10 s; 60 °C for 20 s; 40 cycles to carry out the real-time qPCR reactions according to the instructions. The *gapA* gene was seen as a reference gene. Primers for this study were synthesized by the company (Comate Bioscience Co., Ltd., Changchun, Jilin, China). The primer sequences are provided in Supplementary Table S8. The relative mRNA levels were quantified with the 2^−ΔΔ*CT*^ method. The experiment was conducted in three replicates.

### 4.6. Morphological Observation and Gene Expression Profile of CVCC230 In Vitro

The freshly cultured CVCC230 bacteria were mixed in 5 mL serum-SAPI medium at a ratio of 1:100 and added to NE (100 μM) as the test group (NE group). At the same time, we established a control group without NE. Incubated at 37 °C with shaking at 180 rpm for 14 h, according to the reference with a few modifications [71], the transmission electron microscope (TEM) samples were supposed to be prepared, and the structure of *E. coli* fimbriae was observed under TEM. Centrifuge and collect the 14-h culture; wash and resuspend the thalli in PBS to a concentration of 5 × 10^8^/mL. The cell suspension (10 µL) was coated with a copper grid coated with a thin Formvar film. and air dried. The samples were negatively stained with 2% (wt/vol) uranyl acetate for 1 min after air-drying. Then the electron microscope (HITACHI, Tokyo, Japan, HT7800) was used to photograph.

Total RNA was extracted with TRIzol Reagent (Thermo Fisher Scientific, Waltham, MA, USA), and the transcriptome was analyzed by Shanghai Genesky Biotechnology Co., Ltd. (Shanghai, China). Briefly, check the total RNA quality with an Agilent 2100 Bioanalyzer (Agilent Technologies, Santa Clara, CA, USA). The AgencourtAMPure XP-PCR Purification Beads (Beckman Coulter, Brea, CA, USA) were used to purify and fragment RNA. The SuperScript IV Reverse Transcriptase (Thermo Fisher Scientific, USA) was used to reverse-transcribe first-strand cDNA and then synthesize second-strand cDNA. The Agencourt AMPure XP-PCR Purification Beads were used to purify and subsequently obtain the double-stranded cDNA (ds cDNA). The Agencourt SPRIselect Reagent Kit (Beckman Coulter, USA) was used to screen the original library with a fragment peak at 300 bp. Then the libraries were sequenced using Illumina Hiseq (Illumina, San Diego, CA, USA) with a 2 × 150 bp double-end sequencing strategy.

The KEGG and GO enrichment analyses were performed as described below. Gene Ontology (GO, http://www.geneontology.org/, accessed on 1 January 2019) is an international standard classification system for gene function. After screening the differential genes according to the experimental purpose, studying the distribution of the differential genes in the gene ontology will clarify how the samples in the experiment. KEGG (Kyoto Encyclopedia of Genes and Genomes) is the main public database of experimentally validated classical pathways. The pathway significant enrichment analysis uses the KEGG pathway as a unit and applies a hypergeometric test to identify pathways that are significantly enriched in a given set of genes compared to the whole genomic background.

### 4.7. Statistical Analysis

One-way ANOVA (SPSS 18.0) was used for data comparisons between multiple groups. In each case, *p <* 0.05 was considered statistically significant, and *p <* 0.01 was considered statistically very significant.

## 5. Conclusions

In conclusion, when exposed to catecholamine hormones, ETEC would show an increase in the growth, antimicrobial susceptibility, biofilm formation ability, and gene expression of ten ETEC isolates, and this may be related to the fact that NE regulates the genes associated with it. Our results analyze the mechanism by which NE acts on ETEC through gene expression and could potentially solve the problem of disease exacerbation caused by ETEC under stress conditions.

## Data Availability

The raw data supporting the conclusions of this article will be made available by the authors without undue reservation.

## Supplementary Materials

The supporting information can be downloaded at: https://www.mdpi.com/article/10.3390/ijms242115646/s1.
