# Supplementary material for: Effects of Catecholamine Stress Hormones Norepinephrine and Epinephrine on Growth, Antimicrobial Susceptibility, Biofilm Formation, and Gene Expressions of Enterotoxigenic Escherichia coli
Source: Int J Mol Sci. 2023 Oct 27;24(21):15646. doi: 10.3390/ijms242115646 (PMC10649963; doi:10.3390/ijms242115646)
Supplement: Supplementary file 1 [file ijms-24-15646-s001.zip › ijms-2637781-supplementary.pdf]

International Journal of *Molecular Sciences*

MDPI

Supplementary Materials

Effects of Catecholamine Stress Hormones Norepinephrine and Epinephrine on Growth, Antimicrobial Susceptibility, Biofilm Formation, and Gene Expressions of Enterotoxigenic *Escherichia coli*

Lingdi Niu [1,†], Mingchun Gao [1,2,†], Shanshan Wen [1], Fang Wang [3], Haikun Shangguan [1], Zhiyuan Guo [1], Runxiang Zhang [4,*] and Junwei Ge [1,2,*]

[1] College of Veterinary Medicine, Northeast Agricultural University, Harbin 150030, China
[2] Heilongjiang Provincial Key Laboratory of Zoonosis, Harbin 150030, China
[3] State Key Laboratory of Veterinary Biotechnology, Harbin Veterinary Research Institute, Chinese Academy of Agricultural Sciences, Harbin 150069, China
[4] College of Animal Science and Technology, Northeast Agricultural University, Harbin 150030, China
* Correspondence: zhangrunxiang@neau.edu.cn (R.Z.); gejunwei@neau.edu.cn (J.G.); Tel.: +86-451-5519-0385 (J.G.); Fax: +86-451-5510-3336 (J.G.)
† These authors contributed equally to this work.

Table S1. Impact of NE and Epi on MIC of F4+ ETEC to 14 antibiotics.

isolate		MIC (µg/mL)													
		KAN	AMP	TE	GEN	SXT	APR	STR	FFC	EFT	CIP	TEL	CRO	ENR	PB
52038	C	>256	>256	128	>256	>256	16	128	256	8	8	4	4	128	0.25
	NE	>256	>256	256	>256	>256	32	128	>256	32	8	2	8	256	0.25
	Epi	>256	>256	256	>256	>256	32	256	>256	32	8	4	8	128	0.25
52160	C	128	>256	256	64	>256	64	128	128	64	8	2	8	128	0.5
	NE	>256	>256	256	>256	>256	128	>256	256	128	64	4	8	256	0.5
	Epi	>256	>256	256	>256	>256	256	>256	256	256	64	>4	8	128	0.5
52150	C	256	>256	256	>256	>256	>256	>256	128	32	8	4	8	8	0.5
	NE	>256	>256	256	>256	>256	>256	>256	256	256	64	2	8	8	0.5
	Epi	>256	>256	256	>256	>256	>256	>256	256	256	16	4	8	8	0.5
52130	C	128	>256	256	>256	>256	>256	256	128	64	8	2	4	8	0.5
	NE	>256	>256	256	>256	>256	>256	>256	256	256	32	1	8	8	0.5
	Epi	>256	>256	256	>256	>256	>256	>256	256	256	32	2	8	8	0.5
52011	C	>256	>256	128	>256	8	128	32	128	32	8	2	8	1	0.5
	NE	>256	>256	256	>256	8	256	64	256	64	8	2	8	8	1
	Epi	>256	>256	256	>256	8	256	64	256	32	8	4	8	4	1
52046	C	128	32	32	8	>256	128	8	128	16	8	4	4	0.5	0.25
	NE	>256	>256	64	8	>256	256	256	256	32	64	4	8	32	0.25
	Epi	>256	256	32	8	>256	256	8	256	32	16	>4	8	1	0.25
52164	C	>256	>256	128	>256	>256	256	>256	256	16	8	1	2	16	1
	NE	>256	>256	256	>256	>256	>256	>256	>256	64	8	4	8	128	1
	Epi	>256	>256	128	>256	>256	>256	>256	>256	128	8	>4	8	128	1
52006	C	<256	256	128	32	>256	32	8	32	8	8	4	1	8	1
	NE	>256	>256	>256	32	>256	64	256	128	32	64	4	8	8	1
	Epi	>256	>256	256	64	>256	64	128	128	16	64	4	8	8	1
52156	C	>256	256	256	>256	>256	>256	>256	256	32	8	2	8	>256	1
	NE	>256	>256	>256	>256	>256	>256	>256	>256	64	64	2	8	>256	1
	Epi	>256	>256	>256	>256	>256	>256	>256	>256	64	16	4	8	>256	1
CVCC230	C	256	8	2	4	4	4	2	2	2	1	4	2	1	0.5
	NE	>256	>256	>256	>256	>256	>256	256	256	64	32	8	4	>256	0.5
	Epi	>256	>256	256	128	>256	>256	256	128	32	32	8	4	128	0.5

Note: The C represents the control group. NE and Epi represents the additives are norepinephrine or epinephrine. MIC, minimum inhibitory concentration. KAN, kanamycin; AMP, ampicillin; TE, tetracycline; GEN, gentamicin; SXT, sulfamethoxazole/trimethoprim; APR, apramycin; STR, streptomycin; FFC, florfenicol; EFT, ceftiofur CIP, ciprofloxacin; TEL, tylosin; CRO, ceftriaxone; ENR, enrofloxacin; PB, polymyxin B.

Table S2. Result of partially differential expressed gene associated with growth of CVCC230 strain.

gene_id	CVCC230	NE+CVCC230	baseMean	log2FoldChange	pvalue	type
aceB	5271.2	118816.4	62043	4.4944	0.0084	Up
tdcB	270.1	2207.158	1238.6	3.0305	0.0484	Up
rplW	1128.1	31601.73	16364	4.8078	0.0057	Up
rplC	1863.4	34722.9	18293	4.2197	0.0117	Up
rpsC	1010.8	13360.2	7185.5	3.72425	0.0211	Up
purN	732.5	11974.8	6353.6	4.03085	0.0147	Up
citB	9.5	288.1	148.81	4.9149	0.0086	Up
ygeN	0	60.8	30.42	7.9215	0.0076	Up
gltU	31.3	497.7	264.55	3.9875	0.0187	Up
pcnB	36.8	5851.8	2944.3	7.3116	0.0002	Up
pyrF	6.8	881.1	443.96	7.0131	0.0007	Up
osmY	69466	2764.9	36115	-4.6509	0.0069	Down
sufC	8954.5	799.7	4877.1	-3.4850	0.0280	Down
yqjD	29302	2889.6	16096	-3.3421	0.0329	Down
sufD	16418	1941.8	9179.9	-3.0798	0.0446	Down
ybiI	2160.8	208.9	1184.9	-3.3706	0.0327	Down
uspG	22539	2158.0	12348	-3.3846	0.0313	Down
sufB	18909	1531.3	10220	-3.6262	0.0236	Down
bolA	26780	2935.0	14857	-3.1897	0.0392	Down
yccT	4593.2	562.9	2578.1	-3.0283	0.0478	Down
astC	47022	1143.5	24083	-5.3617	0.0029	Down

Table S3. Result of differential expressed gene associated with drug resistance of CVCC230 strain.

gene_id	CVCC230	NE+CVCC230	baseMean	log2FoldChange	pvalue	type
emrY	17.7	205.9	111.8	3.5378	0.0372	Up
yhdT	12.2	174.4	93.36	3.8287	0.0298	Up
mepS	414.7	5623.8	3019.2	3.7613	0.0204	Up
astE	23136	1227.1	12181	-4.2368	0.0115	Down
kdpE	1566.1	181.8	873.9	-3.1068	0.0447	Down
hdeD	30901	3439.3	17170	-3.1674	0.0402	Down
yjdN	24656	2316.4	13486	-3.412	0.0303	Down
yfcG	3752.9	412.6	2082.8	-3.184	0.04	Down
glsA	2654.7	206.7	1430.7	-3.682	0.0226	Down
treF	10265	966.1	5615.8	-3.409	0.0305	Down
nhoA	2743.4	338.6	1541	-3.018	0.0487	Down

Table S4. Result of partially differential expressed gene associated with biofilm formation of CVCC230 strain.

gene_id	CVCC230	NE+CVCC230	baseMean	log2FoldChange	pvalue	type
narJ	987.7	8661.5	4824.6	3.1324	0.04	Up
narI	466.5	3956.9	2211.7	3.0842	0.045	Up
lsrC	46.3	952.2	499.2	4.3595	0.0112	Up
malF	418.8	6504.9	3461.8	3.9571	0.0162	Up
malK	159.6	6590.7	3375.1	5.3677	0.0030	Up
malE	214.2	14072.7	7143.4	6.0379	0.0012	Up
adeP	791.2	7430	4110.6	3.2311	0.0377	Up
modC	632.9	12106	6369.5	4.2573	0.0112	Up
modB	548.4	11639	6093.7	4.4075	0.0094	Up
modA	556.6	27723.9	14140	5.6383	0.002	Up
ompF	13311	116544	64927	3.13	0.042	Up
yibH	37168	1207.3	19188	-4.9442	0.0049	Down
yqaE	403.8	46.9	225.3	-3.1055	0.0494	Down
yhjD	5934.2	464	3199.1	-3.6768	0.0224	Down
yjcC	10842	1091.4	5967.1	-3.3123	0.0341	Down
yqjE	16834	1617.8	9225.9	-3.3792	0.0315	Down
uspB	27234	911.9	14073	-4.9004	0.0051	Down
ygiW	4163.5	400.2	2281.8	-3.3788	0.0319	Down
hspQ	28993	2869.1	15931	-3.3371	0.0331	Down
yejB	939.9	12.4	476.19	-6.2369	0.0012	Down

Table S5. Result of differential expressed gene associated with virulence of CVCC230 strain.

gene_id	CVCC230	NE+CVCC230	baseMean	log2FoldChange	pvalue	type
gspJ	10.9	139.2	75.1	3.67	0.04	Up
tfaQ	5.4	473.5	239.5	6.44	0.00	Up
lysV	13.6	210.4	112	3.95	0.02	Up
flhD	536.1	5302	2919.1	3.31	0.03	Up
flhC	1349.2	12490	6920	3.21	0.04	Up
fliS	1.4	45.4	23.4	5.06	0.04	Up
entB	17.7	947.1	482.4	5.74	0.00	Up
fepA	714.8	7059	3886.9	3.30	0.03	Up
purL	702.5	34888.6	17795.6	5.63	0.00	Up
estB	635	1789	1212	1.49	0.02	Up
elt	375	3231	1803	3.03	0.01	Up
yehB	43.6	0.00	21.8	-8.34	0.01	Down
relE	8044.7	940.5	4492.6	-3.1	0.04	Down
cbpA	14302.3	1797.4	8049.8	-2.9	0.05	Down
pabA	6956	628.9	3792.5	-3.5	0.03	Down

Table S6. The information of strains.

Strains	Genetic type	Biofilm	Virulence	Antimicrobial resistance								
				GEN	TE	CRO	TEL	AMP	CIP	SXT	STR	COL
52038	A	-	*feaG, STa, STb*	R	R	R	R	R	S	R	R	S
52160	A	+	*feaG, STa, STb*	R	R	S	R	R	S	R	R	S
52150	B1	+++	*feaG, Stx2e, STa, STb*	R	R	R	R	R	R	R	R	S
52130	A	+++	*feaG, EAST1, Stx2e, STa, STb*	R	R	R	R	R	R	R	R	S
52011	A	+++	*feaG, STa*	R	R	R	I	R	S	R	R	S
52046	A	+	*feaG, STb*	R	R	S	R	R	S	R	R	S
52164	A	++	*feaG*	R	R	R	R	R	S	R	R	S
52006	B2	+++	*feaG, EAST1, STa*	R	R	R	R	R	I	R	R	S
52156	A	-		R	R	R	R	R	R	R	R	R
CVCC230	/	-	*feaG, STb, LT*	S	S	S	S	R	S		S	S

Note: "-" means non-adherent; "+" means weakly-adherent; "++" means moderately adherent; "+++" means strongly adherent. GEN, gentamicin; TE, tetracycline; CRO, ceftriaxone; TEL, tylosin; AMP, ampicillin; CIP, ciprofloxacin; SXT, sulfamethoxazole/trimethoprim; STR, streptomycin; COL, colistin.

Table S7. Primer sequences for *virulence genes* identification and genotype in ETEC strains by PCR.

Gene	Sequence (5'–3')	Amplified product (bp)	temperature (◦C)
feaG	F: GGTGATTTCAATGGTTCG<br>R: ATTGCTACGTTCAGCGGAGCG	764	63
STa,	F: TCCGTGAAACAACATGACGG<br>R: ATAACATCCAGCACAGGCAG	244	63
STb	F: GCCTATGCATCTACACAATC<br>R: TGAGAAATGGACAATGTCCG	279	63
Stx2e	F: AATAGTATACGGACAGCGAT<br>R: TCTGACATTCTGGTTGACTC	733	63
EAST1,	F: TCGGATGCCATCAACACAGT<br>R: GTCGCGAGTGACGGCTTTGTAG	125	63
LT	F: ATTTACGGCGTTACTATCCTC<br>R: TTTTGGTCTCGGTCAGATATG	275	49
gadA	F: GATGAAATGGCGTTGGCGCAAG<br>R: GGCGGAAGTCCCAGACGATATCC	373	65
chuA	F: ATGATCATCGCGGCGTGCTG<br>R: AAACGCGCTCGCGCCTAAT	281	65
yjaA	F: TGTTCGCGATCTTGAAAGCAAACGT<br>R: ACCTGTGACAAACCGCCCTCA	216	65
TSPE4.C2	F: GCGGGTGAGACAGAAACGCG<br>R: TTGTCGTGAGTTGCGAACCCG	152	65

Table S8. Primer sequences for qPCR.

Gene	Forward	Reverse
gapA	TCCGTGCTGCTCAGAAACG	CACTTTCTTCGCACCAGCG
feaG	AATGCATCTTATGCCGGTG	TCTTTGAATCTGTCCGAGAATATC
estB	TGCCTATGCATCTACACAAT	CTCCAGCAGTACCATCTCTA
estA	GTGGTCCTGAAAGCATGAATAG	AATAGCACCCGGTACAAGCA
elt	TCTCTATGTGCATACGGAGC	CCATACTGATTGCCGCAAT
aceB	GTTGGTCTGAACTGCGGTCGTTGG	TCCATCGTCACTGCCTGTCTGTCTG
tdcB	GTTCACGGCATGGCGGCTTCTT	GTCTTCGCTGACCAGCACGATGTC
rplW	AACGTCACGGACAGCGTATCGG	TTACTCAGCGCCGCCAACGAAG
emrY	CGCTCCGATATGTGGGCCGATATTG	AGCCACCAACACCGAGCACTAACA
yhdT	AGCCGCTTACTTATCTGGCGTTGC	GCCCAGCACAGTCCAATAAACAGCA
narJ	CGAGCAGCACGGCTTGCAGTTA	ACCTTCCACGGCTTCGCTTTGC
lsrC	TTCTGCTGGCGGTGCTGGTGTT	ACGGATGGCGGTGGCGTCATAA
malF	AACCGCTGACGCCGCTGATGAT	ATTGCTGCCGCCAGACCGAAGT
gspJ	CTGTGGATGGCAGGAAGGATCAACC	CGTCTGAGCTGGCGACTCTTCTCT
tfaQ	TCCGCCTCATACAGGTCTGCCA	GTCGCCGGAAGCCACGTCATAA
flhD	GGCACTGACTCTTCCGCAAATGGTT	TTCAGCAAGCGTGTTGAGAGCATGA

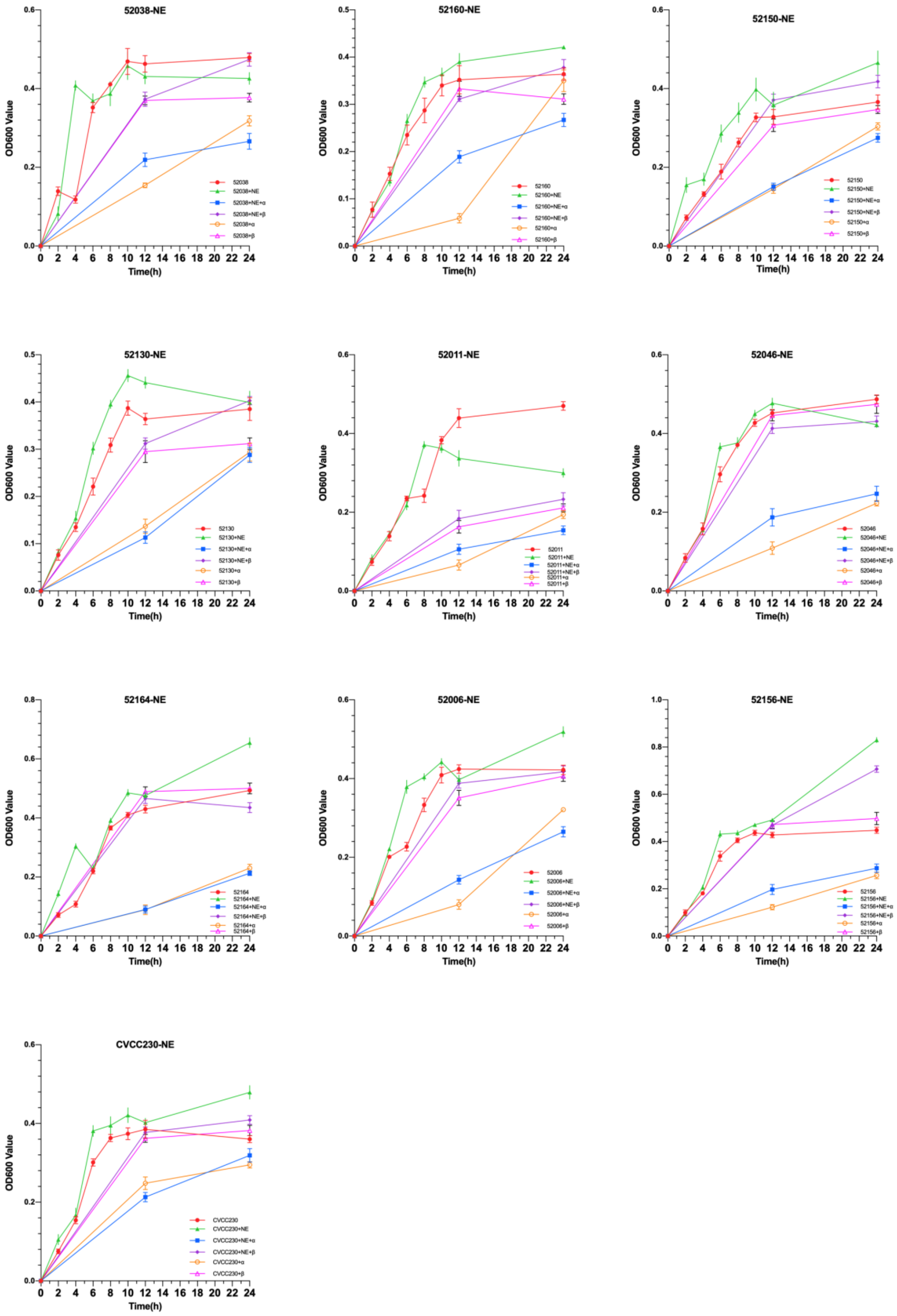

Figure S1. Exploring the effects of the α-adrenergic receptor antagonist (phentolamine) and β-adrenergic receptor antagonist (propranolol) on the growth of ten ETEC strains promoted by NE.

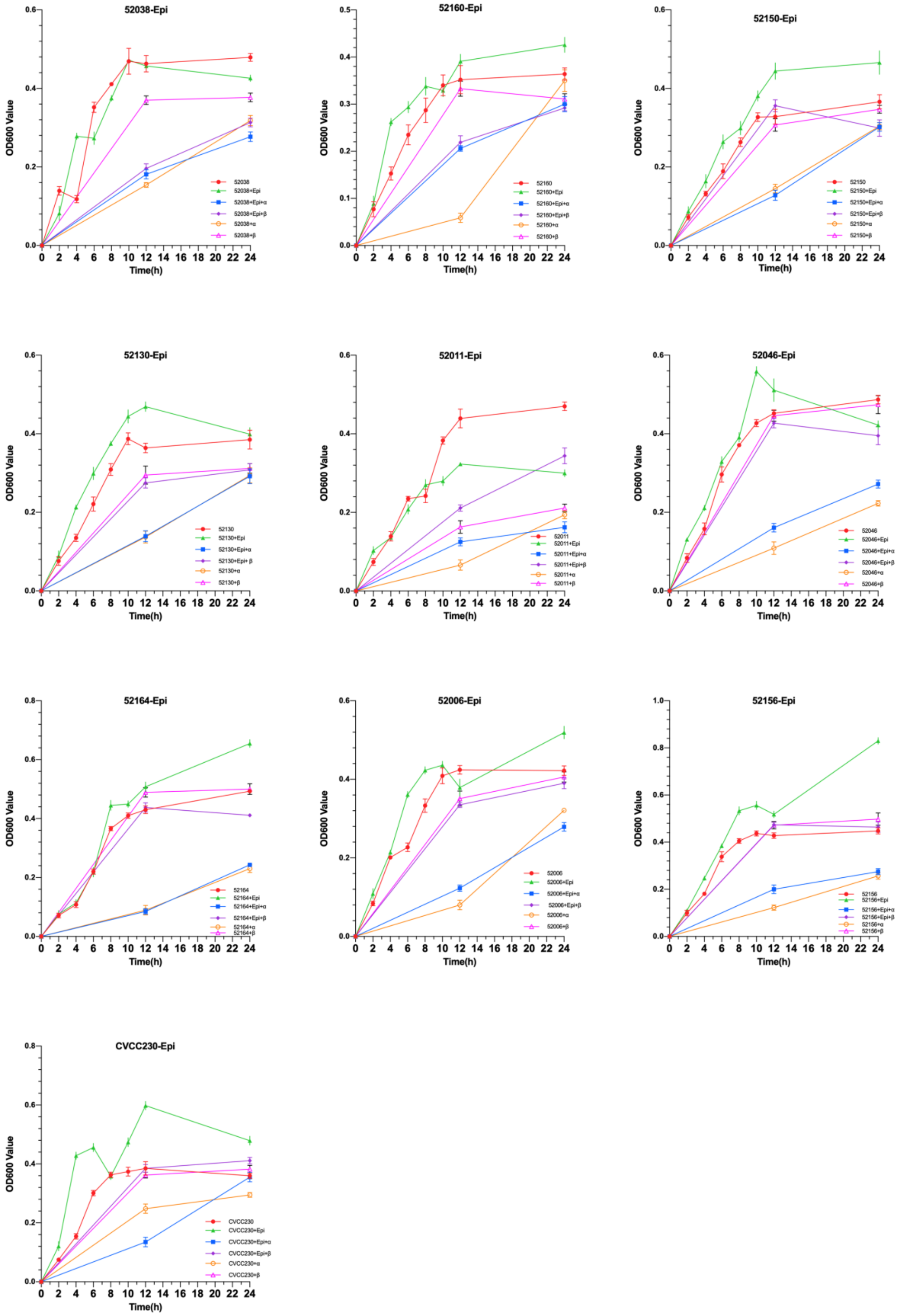

Figure S2 Exploring the effects of the α-adrenergic receptor antagonist (phentolamine) and β-adrenergic receptor antagonist (propranolol) on the growth of ten ETEC strains promoted by Epi.
